# Iranian nurses’ perceptions about using physical restraint for hospitalized elderly people: a cross-sectional descriptive-correlational study

**DOI:** 10.1186/s12877-020-01636-2

**Published:** 2020-07-06

**Authors:** Azam Sharifi, Narges Arsalani, Masoud Fallahi-Khoshknab, Farahnaz Mohammadi-Shahbolaghi, Abbas Ebadi

**Affiliations:** 1 Nursing Department, University of Social Welfare and Rehabilitation Sciences, Tehran, Iran; 2 Nursing School, Baqiyatallah University of Medical Sciences, Tehran, Iran; 3 Behavioral Sciences Research Center, Life Style Institute, Baqiyatallah University of Medical Sciences, Tehran, Iran

**Keywords:** Physical restraint, Nurse, Elderly, Hospital

## Abstract

**Background:**

Using physical restraint (PR) for hospitalized elderly people is a major nursing challenge. It is associated with different physical and mental complications and ethical dilemmas, though many nurses still use it to ensure patient safety. Nurses’ perceptions are one of the most important factors affecting PR use. This study aimed to evaluate Iranian nurses’ perceptions about PR use for hospitalized elderly people.

**Methods:**

This cross-sectional descriptive-correlational study was conducted from July to December 2019. Participants were 270 hospital nurses who were purposively recruited from intensive care units and medical and surgical wards of three teaching hospitals in Kermanshah, Iran. Data were collected using a demographic questionnaire and the Perceptions of Restraint Use Questionnaire (PRUQ). The SPSS software (v. 23.0) was used for data analysis through the independent-sample *t* test, the one-way analysis of variance, and the multiple regression analysis.

**Results:**

The total mean score of PRUQ was 4.08 ± 0.12 in the possible range of 1–5. The most important reasons for PR use were to prevent patients from falling out of bed and to prevent them from pulling out catheters. The total mean score of PRUQ had significant relationship with participants’ age, work experience, and history of receiving PR-related educations (*P* < 0.05), but had no significant relationship with their gender, educational degree, and affiliated hospital ward (*P* > 0.05).

**Conclusion:**

This study suggests that nurses attach high importance to PR use for hospitalized elderly people. Healthcare policy-makers at national and hospital levels are recommended to provide nurses with PR-related educations in order to reduce the rate of PR-related complications.

## Background

Patient safety is a main component of healthcare quality [1]. Ensuring patient safety is among the basic rights of patients and one of the main goals of nursing care [2, 3].

Restraint is one of the methods for ensuring patient safety [4–6]. Restraint refers to any medication or device used to restrict patients’ voluntary movements in order to prevent injuries to patients and others [7, 8]. There are two main types of restraint, namely chemical and physical. Chemical restraint is to calm patients, lower the level of their consciousness, and reduce their responsiveness to environmental stimuli through sedative agents. In chemical restraint, sedative agents (including opioids, benzodiazepines, and muscle relaxants) are used to induce tranquility and pain relief, reduce intracranial pressure, and prevent the accidental removal of catheters [8–10]. Physical restraint (PR) refers to “any action or procedure that prevents a person’s free body movement to a position of choice and/or normal access to his/her body by the use of any method, attached or adjacent to a person’s body that he/she cannot control or remove easily” [11]. PR devices include wrist, ankle, chest, and waist restraints and bed rails [6, 11].

The prevalence of PR use among hospitalized patients is 10–75% [12–15]. This prevalence is three times greater among elderly people [16, 17]. This high prevalence may be related to the high hospitalization rate among elderly people secondary to their affliction by different chronic conditions [18, 19]. Hospitalization of elderly people is associated with different adverse events and safety issues because most of them have poor health status, use multiple medications, may suffer from cognitive or functional disorders, and hence, are at risk for fall and injury to self or others [17, 20–22]. Therefore, nurses often use PR to restrict their movements and ensure their safety [23, 24].

Nurses have many reasons for PR use. These reasons include ensuring patient safety, preventing treatment discontinuation, preventing patient fall, controlling agitated or restless patients, protecting patients and others against injuries, creating a safe environment, and overcoming the problems associated with staff shortage [3, 4, 6].

Although PR is used to ensure patient safety, there is limited evidence concerning its effectiveness [25]. Studies show that PR can endanger patient safety and cause different physical and mental complications. Physical complications include pressure ulcer, contracture, physical and cognitive dysfunction, prolonged hospital stay, increased likelihood of patient fall, asphyxia, and death due to strangulation [14, 26–28]. PR-associated mental complications include anger, frustration, aggression, fear, anxiety, depression, and reduced self-confidence [29–32]. Besides, PR use is associated with ethical dilemmas because it contradicts patients’ right to have autonomy and may negatively affect their dignity [33, 34]. PR use for hospitalized elderly people can also delay recovery [5, 19, 35].

Nurses have significant role in decision making about PR use [3, 4, 36–40]. One of the factors which may require nurses to use PR is their perceptions about PR use [2, 24, 41, 42]. Studies showed that nurses’ decision about PR use is greatly affected by their perceptions [3, 6, 41–43]. A mixed-method study also showed that a sense of security, heavy workload, and staff shortage can affect nurses’ perceptions about PR use [6]. Other factors affecting nurses’ perceptions about PR use include patients’ characteristics, nurses’ knowledge and attitudes, cultural factors, and professional regulations [4, 6, 31, 35, 44–46]. There are strict regulations on PR use in some countries [4, 20, 31, 42]; however, there is no clear guideline for PR use in healthcare settings in Iran.

Some studies showed that despite the wide use of PR, nurses have limited knowledge about appropriate PR use [40, 47].

Although different studies evaluated nurses’ perceptions about PR use in different countries [2, 6, 24, 31, 41, 42], there are limited data, if any, about Iranian nurses’ perceptions about PR use. The present study was conducted to address this gap. The aim of the study was to evaluate nurses’ perceptions about PR use for hospitalized elderly people. Understanding these perceptions can form a basis for developing and using interventions to reduce PR use.

## Methods

This cross-sectional descriptive-correlational study was conducted from July to December 2019 in three teaching hospitals in Kermanshah, Iran. Kermanshah is a large city in the west of Iran with three general teaching hospitals and several specialty teaching hospitals (including cardiac, psychiatric, maternity, and children’s hospitals). Study setting was the three general teaching hospitals in the city and study population consisted of all 688 nurses who worked in these hospitals. Using the Cochran formula and with a confidence level of 95% and a *d* of 0.05, sample size was calculated to be 246. Yet, considering a probable attrition rate of 10% [48], sample size was increased to 270. As the number of nurses in the intensive care units, medical care wards, and surgical care wards in the study setting was almost the same, we selected ninety nurses from each of these wards to the study. Sampling was performed purposively. Eligibility criteria were work experience of more than 1 year in adult intensive care units or medical-surgical wards and recent use of PR for elderly people. Nurses who worked in other hospital wards (such as pediatric wards) and those who had managerial positions (such as head nurses) were not included in the study.

### Data collection

Data collection instruments were a demographic questionnaire (with items on gender, age, educational degree, work experience, and self-report history of receiving PR-related educations at university or during professional practice) and the Perceptions of Restraint Use Questionnaire (PRUQ) (Additional file 1). PRUQ was developed by Evans and Strumpf in 1988 and was revised in 1993. As a self-report questionnaire, PRUQ is used to assess healthcare providers’ perceptions about the relative importance of PR use for elderly people [49, 50]. It has seventeen items which are scored on a five-point scale from 1 (“Not important”) to 5 (“Very important”), resulting in a possible total score of 17–85 which also can be reported in the range of 1–5. Higher scores show greater importance of PR use. Sharifi et al. [51] evaluated and confirmed the validity and the reliability of the Persian version of this questionnaire. Exploratory factor analysis in their study showed that the Persian PRUQ had three main dimensions, namely, “prevention of fall”, “prevention of treatment discontinuation”, and “creation of a safe environment”. Confirmatory factor analysis also confirmed the appropriateness of this three-factor structure. Reliability assessment in that study also showed that the test-retest intraclass correlation coefficient, the Cronbach’s alpha, and the construct reliability of the Persian PRUQ were 0.86 (95% confidence interval: 0.74–0.93; *P* < 0.001), 0.82, and more than 0.7, respectively. For data collection, participants were provided with the questionnaire and were asked to complete it at their earliest convenience. To minimize the frequency of the missing data, we provided participants with clear explanations about completing the questionnaire and answered their questions.

### Data analysis

Data were analyzed using the SPSS software (v. 23.0). The measures of descriptive statistics as well as the independent-sample *t* test, the one-way analysis of variance, and the multiple regression analysis were used to describe and analyze the data at a significance level of less than 0.05. In regression analysis, numerical variables (such as age and work experience) were entered into the model at ratio level, while categorical variables (such as gender and educational degree) were entered at nominal and ordinal levels.

### Ethical considerations

The Research Ethics Committee of the University of Social Welfare and Rehabilitation Sciences, Tehran, Iran, approved the study (IR.USWR.REC.1398.121). Nurses were informed about the study aim, confidentiality of their data, and voluntariness of participation, and then, written informed consent was obtained from all of them.

## Results

In total, 270 nurses participated in the study. Response rate was 100% and there were no missing data. Most of them were female (78.5%), had Bachelor’s degree in nursing (58.52%), and reported no history of receiving PR-related educations (88.5%). The means of their age and work experience were 35.91 ± 6.31 and 8.87 ± 5.48 years, respectively.

The total mean score of PRUQ was 4.08 ± 0.12 in the possible range of 1–5. It had significant relationship with participants’ age, work experience, and history of receiving PR-related educations (*P* < 0.05), but had no significant relationship with their gender, educational degree, and affiliated hospital ward (*P* > 0.05; Table 1).

**Table 1:** Participants’ demographic characteristics and total mean score of PRUQ

Characteristics		N (%)	PRUQ score (Mean ± SD)
**Gender**	Female Male Test results^a^	212 (78.5) 58 (21.5)	4.09 ± 0.12 4.05 ± 0.14 *t* = − 2.383, *P* = 0.338
**Age (Years)**	20–29 30–39 ≥ 40 Test results^b^	68 (25.1) 116 (43.0) 86 (31.9)	3.92 ± 0.76 4.07 ± 0.48 4.21 ± 0.59 F = 451.410, *P* = 0.001
**Educational degree**	Bachelor’s Master’s Test results^a^	158 (58.52) 112 (41.48)	4.09 ± 0.12 4.07 ± 0.13 *t* = −1.371, *P* = 0.172
**Work experience (Years)**	1–5 6–10 > 10 Test results^b^	75 (27.8) 114 (42.2) 81 (30.0)	3.94 ± 0.87 4.08 ± 0.55 4.21 ± 0.71 F = 29.677, P = 0.001
**History of receiving PR-related education**	Yes No Test results^a^	31 (11.5) 239 (88.5)	3.86 ± 0.51 4.11 ± 0.89 *t* = −13.562, *P* = 0.001
**Affiliated hospital ward**	Intensive care unit Medical ward Surgical ward Test results^b^	90 (33.3) 90 (33.3) 90 (33.3)	4.09 ± 0.12 4.06 ± 0.13 4.08 ± 0.13 F = 0.929, *P* = 0.396

^a^The results of the independent-sample *t* test

^b^The results of the one-way analysis of variance

The multiple regression analysis through the Enter method was used to determine the predictors of the PRUQ score. Its results revealed that the significant predictors of the PRUQ score were age, work experience, and history of receiving PR-related educations. These three variables explained 82% of the total variance of the PRUQ score (*P* < 0.05; Table 2).

**Table 2:** The results of multiple regression analysis for predicting the total mean score of PRUQ based on demographic characteristics

Predictors variable	B	SE	Beta	***t***	***P***
Constant	3.628	0.025	–	147.772	0.001
Gender	0.004	0.008	0.013	0.502	0.616
Age	0.101	0.012	0.612	8.692	0.001
Educational degree	0.002	0.007	0.007	0.285	0.776
Work experience	0.023	0.011	0.142	2.095	0.037
History of receiving PR-related education	0.101	0.012	0.260	8.603	0.001

*R* = 0.907

*R*^*2*^ = 0.823

Adjusted *R*^*2*^ = 0.820

Participants’ most important reasons for using PR were “Protecting an older person from falling out of bed”, “Preventing an older person from pulling out a feeding tube”, and “Preventing an older person from pulling out a catheter”, respectively. The least important reasons were “Preventing an older person from taking things from others”, “Keeping a confused older person from bothering others”, and “Preventing an older person from wandering”. The most important reasons for using PR in intensive care units were the same as medical wards and included “Protecting an older person from falling out of bed”, “Preventing an older person from pulling out a feeding tube”, and “Preventing an older person from pulling out a catheter”, respectively. The most important reasons for using PR in surgical wards were “Preventing an older person from pulling out a feeding tube”, “Protecting an older person from falling out of bed”, and “Preventing an older person from pulling out a catheter”, respectively. The total mean score of PRUQ among nurses in intensive care units was insignificantly greater than nurses in other hospital wards (*P* = 0.396). The only difference among nurses in different hospital wards was related to item 9, i.e. “Substituting for staff observation” (*P* = 0.001). The results of the Tukey’s post hoc test indicated that the mean score of this item among nurses in intensive care units was significantly greater than nurses in medical and surgical wards (*P* = 0.001); however, there was no significant difference between nurses in medical and surgical wards respecting the mean score of this item (*P* = 0.992; Table 3).

**Table 3:** The total mean scores of PRUQ and its items according to participants’ affiliated hospital ward

PRUQ items	Intensive care unit (***N*** = 90) Mean ± SD	Medical ward (***N*** = 90) Mean ± SD	Surgical ward (***N*** = 90) Mean ± SD	Total (***N*** = 270) Mean ± SD	***P*** value^*^
1. Protecting an older person from:					
a. Falling out of bed	4.92 ± 0.27	4.86 ± 0.34	4.84 ± 0.36	4.88 ± 0.33	0.262
b. Falling out of a chair	3.56 ± 0.49	3.46 ± 0.50	3.41 ± 0.49	3.48 ± 0.50	0.107
c. Unsafe ambulation	4.61 ± 0.49	4.58 ± 0.58	4.56 ± 0.58	4.59 ± 0.55	0.864
2. Preventing an older person from wandering	2.87 ± 0.33	2.95 ± 0.21	2.89 ± 0.32	2.91 ± 0.29	0.151
3. Preventing an older person from taking things from others	2.58 ± 0.50	2.52 ± 0.51	2.66 ± 0.47	2.59 ± 0.49	0.140
4. Preventing an older person from getting into dangerous places or supplies	3.81 ± 0.39	3.84 ± 0.36	3.87 ± 0.34	3.84 ± 0.37	0.594
5. Keeping a confused older person from bothering others	2.90 ± 0.32	2.90 ± 0.30	2.88 ± 0.32	2.89 ± 0.31	0.963
6. Preventing an older person from:					
a. Pulling out a catheter	4.89 ± 0.32	4.79 ± 0.41	4.81 ± 0.39	4.83 ± 0.38	0.174
b. Pulling out a feeding tube	4.90 ± 0.30	4.82 ± 0.38	4.88 ± 0.34	4.87 ± 0.34	0.289
c. Pulling out an IV	4.83 ± 0.40	4.73 ± 0.65	4.74 ± 0.57	4.77 ± 0.55	0.098
d. Breaking open sutures	4.70 ± 0.59	4.54 ± 0.65	4.64 ± 0.64	4.63 ± 0.66	0.246
e. Removing a dressing	4.63 ± 0.58	4.59 ± 0.60	4.61 ± 0.58	4.61 ± 0.58	0.882
7. Providing quiet time or rest for an overactive older person	3.51 ± 0.50	3.53 ± 0.62	3.56 ± 0.56	3.53 ± 0.56	0.802
8. Providing for safety when judgment is impaired	4.72 ± 0.45	4.57 ± 0.58	4.55 ± 0.60	4.61 ± 0.55	0.076
9. Substituting for staff observation	4.04 ± 0.68	4.42 ± 0.56	4.43 ± 0.56	4.31 ± 0.62	0.001
10. Protecting staff or other patients from physical abusiveness/combativeness	3.39 ± 0.57	3.53 ± 0.54	3.51 ± 0.67	3.47 ± 0.60	0.242
11. Managing agitation	4.70 ± 0.46	4.57 ± 0.52	4.55 ± 0.49	4.61 ± 0.51	0.094
Total PRUQ mean score	4.09 ± 0.12	4.06 ± 0.13	4.08 ± 0.13	4.08 ± 0.12	0.396

^*^The results of the one-way analysis of variance

## Discussion

This study assessed nurses’ perceptions about using PR for elderly people hospitalized in intensive care units and medical and surgical wards. The total mean score of PRUQ was 4.08 ± 0.12 (in the possible range of 1–5), denoting that nurses considered PR use very important for care delivery to hospitalized elderly people. The high total score of PRUQ can also denote the wide use of PR [41, 43]. Previous studies reported PR use as a simple and effective method for ensuring patient safety and preventing treatment discontinuation [3, 24, 39]. Yet, PR is considered as a dangerous intervention due to its physical, mental, and ethical complications and hence, should be used only in specific situations [2, 52, 53]. The total mean score of PRUQ in the present study was almost the same as the findings of a study in Turkey [2] which reported a score of 4.14, but was much larger than the scores reported in studies in Ireland [31] and the United States [42] which both were equal to 2.8. This discrepancy may be due to the different PR use guidelines and regulations used in different healthcare settings. Evidence shows that effective PR-related legislations and periodical supervision of nurses’ PR use can reduce their tendency for using PR [3, 4, 31]. There is no clear guideline for PR use in healthcare settings in Iran and hence, most nurses are unaware of appropriate PR use. On the other hand, nursing staff shortage in Iran makes nurses consider PR as an effective method for reducing their workload [40, 47]. Alleviating staff shortage, providing quality PR-related educations to nurses, and developing clear PR use guidelines are needed to reduce the inappropriate use of PR by nurses.

Study findings showed that nurses’ most important reasons for using PR for hospitalized elderly people were to prevent them from falling out of bed and to prevent them from pulling out their feeding tubes and catheters. Several earlier studies also reported the same finding [2, 6, 42]. However, some studies showed that PR use does not necessarily reduce the risks of falling out of bed and accidental removal of catheters by patients [30, 43, 54]. Therefore, alternative methods should be used to reduce inappropriate use of PR. These methods include continuous patient monitoring, supporting and reassuring patients to reduce their anxiety, fulfilling their physical needs, increasing their mobility, involving their family members in the process of care delivery, improving environmental safety, using alarm systems, and covering catheters to make them invisible [20, 24, 26, 35, 55]. Some studies also recommended the early removal of catheters immediately after procedures and the limited use of invasive procedures in order to reduce the need for PR [17, 20, 30].

We also found that nurses with older age and greater work experience obtained significantly higher PRUQ scores. These findings are in line with the findings of two earlier studies [2, 56]. Evidence shows that nurses’ tendency for using invasive procedures during patient care increases with age and work experience probably due to the fact that they become more experienced and more courageous over time [57, 58]. Moreover, nurses’ empathy with patients may reduce over time due to problems such as unresolved fatigue and job burnout [56], resulting in their progressive indifference to the pain and suffering associated with invasive procedures. The experiences and preferences of older nurses may affect younger nurses and increase their tendency for using PR. Therefore, periodical educations about appropriate PR use are needed, particularly for older nurses.

Study findings also revealed that nurses with the history of receiving PR-related educations obtained lower PRUQ scores. In other words, they attached lower importance to PR use. This finding implies that receiving quality educations about PR, its complications, and its alternatives can reduce its use by nurses. Several former studies also reported the same finding [24, 26, 53]. Moreover, we found that nurses’ PRUQ score had no significant relationship with their educational degree. Contrarily, a former study reported that nursing assistants obtained higher PRUQ scores than registered nurses and attributed this finding to nursing assistants’ lower information about the complications of PR use [24]. This difference between these two studies may be due to the fact that appropriate PR use is not adequately addressed in different nursing education programs in Iran. Two studies in Iran [40, 47] showed that nurses had limited knowledge about appropriate PR use and highlighted the necessity of providing quality PR-related educations at different levels of nursing education.

Our findings also indicated that while nurses in intensive care units obtained higher PRUQ scores than nurses in medical and surgical wards, this difference was not statistically significant. Previous studies [2, 6, 30] reported the higher prevalence of PR use in intensive care units. Patients in intensive care units are critically-ill and are connected to different devices and systems. Therefore, they are more at risk for injuries and more likely to receive PR.

We also found that compared with nurses in intensive care units, their counterparts in medical and surgical wards assigned higher importance to the substitution of PR use for staff observation. This finding may be due to the limited number of beds in intensive care units for elderly patients in the study setting which resulted in their hospitalization in general hospital wards. Nurses in medical and surgical wards are more likely to substitute PR for staff observation due to low nurse-patient ratio and lack of patient monitoring systems in their wards.

### Limitations

One of the study limitations was purposive sampling which limits the generalizability of the findings.

## Conclusion

This study concludes that hospital nurses consider PR use as an important procedure for preventing patients from falling out of bed and removing catheters and tubes. The findings of the present study can be used as a guideline to provide nurses with in-service PR-related educations. Moreover, the findings highlight the need for improving staffing level, providing necessary equipment for using PR alternatives, and developing culturally-appropriate PR use guidelines and protocols.

## Supplementary information

**Additional file 1.** Data collection instruments. Perceptions of Restraint Use Questionnaire (PRUQ) and a demographic questionnaire.

## Data Availability

Data and material are available by the corresponding author on reasonable request. In order to access the raw data, it is necessary to obtain the permission of the University Research Vice-Chancellor.

## Abbreviations

PR: Physical Restraint
PRUQ: Perceptions of Restraint Use Questionnaire
SPSS: Statistical Product and Service Solutions
