# Supplementary material for: Iranian nurses’ perceptions about using physical restraint for hospitalized elderly people: a cross-sectional descriptive-correlational study
Source: BMC Geriatr. 2020 Jul 6;20:233. doi: 10.1186/s12877-020-01636-2 (PMC7339549; doi:10.1186/s12877-020-01636-2)
Supplement: Supplementary file 1 — Additional file 1. Data collection instruments. Perceptions of Restraint Use Questionnaire (PRUQ) and a demographic questionnaire. [file 12877_2020_1636_MOESM1_ESM.docx]

**Data collection instruments**

(English version)

DATE___/___/___

**Perceptions of Restraint Use Questionnaire (PRUQ)^*^**

In caring for the older adult, physical restraints are sometimes used. Such restraints include vests, belts or sheet ties, crotch or diaper restraints, ankle or wrists ties, hand mitts, or locked geriatric chairs with fixed tray tables.

Following are reasons sometimes given for restraining older people. In general, how important do you believe the use of physical restraints are for each reason listed? (please circle the number that represents your choice).

| Most important |  | Some what important |  | Not at all important | PRUQ items |
| --- | --- | --- | --- | --- | --- |
|  | | | | | 1.Protecting an older person from: |
| 5 | 4 | 3 | 2 | 1 | a. Falling out of bed |
| 5 | 4 | 3 | 2 | 1 | b. Falling out of a chair |
| 5 | 4 | 3 | 2 | 1 | c. Unsafe ambulation |
| 5 | 4 | 3 | 2 | 1 | 2. Preventing an older person from wandering |
| 5 | 4 | 3 | 2 | 1 | 3. Preventing an older person from taking things from others |
| 5 | 4 | 3 | 2 | 1 | 4. Preventing an older person from getting into dangerous places or supplies |
| 5 | 4 | 3 | 2 | 1 | 5. Keeping a confused older person from bothering others |
|  | | | | | 6. Preventing an older person from: |
| 5 | 4 | 3 | 2 | 1 | a. Pulling out a catheter |
| 5 | 4 | 3 | 2 | 1 | b. Pulling out a feeding tube |
| 5 | 4 | 3 | 2 | 1 | c. Pulling out an IV |
| 5 | 4 | 3 | 2 | 1 | d. Breaking open sutures |
| 5 | 4 | 3 | 2 | 1 | e. Removing a dressing |
| 5 | 4 | 3 | 2 | 1 | 7. Providing quiet time or rest for an overactive older person |
| 5 | 4 | 3 | 2 | 1 | 8. Providing for safety when judgment is impaired |
| 5 | 4 | 3 | 2 | 1 | 9. Substituting for staff observation |
| 5 | 4 | 3 | 2 | 1 | 10. Protecting staff or other patients from physical abusiveness/combativeness |
| 5 | 4 | 3 | 2 | 1 | 11. Managing agitation |

**^*^Source:** Evans LK, Strumpf NE. Frailty and physical restraint. In Aging and Musculoskeletal Disorders. New York: Springer; 1993. Available from: <https://ltctoolkit.rnao.ca/sites/default/files/resources/RNAO_Safety_Alternative_App_to_Use_Restraints_AppK_pg108_110.pdf>. Accessed 14 Aug 2018.

**Demographic questionnaire**

1. Gender: ❑ Female ❑ Male
2. Age: -------- years old
3. Type of Unit: ❑ Medical ward ❑ Surgical ward ❑ Intensive care unit
4. Total length of work experience: -------- years
5. Educational degree: ❑ MSN ❑ BSN
6. History of receiving PR-related educations at university or during professional practice: ❑ Yes ❑ No
